# IPR treatment and attachments design in clear aligner therapy and risk of open gingival embrasures in adults

**DOI:** 10.1186/s40510-022-00452-1

**Published:** 2023-01-09

**Authors:** Yubohan Zhang, Xu Wang, Jihong Wang, Jie Gao, Xulin Liu, Zuolin Jin, Yanning Ma

**Affiliations:** 1 State Key Laboratory of Military Stomatology and National Clinical Research Center for Oral Diseases and Shaanxi Clinical Research Center for Oral Diseases, Department of Orthodontics, School of Stomatology, Air Force Medical University, Xi’an, 710032 China; 2 State Key Laboratory of Cancer Biology, National Clinical Research Center for Digestive Diseases and Xijing Hospital of Digestive Diseases, Fourth Military Medical University, Xi’an, Shaanxi China; 3 The First People’s Hospital of Xianyang, Xianyang City, 712000 China; 4 Shanxi Medical University School and Hospital of Stomatology, Taiyuan, 030001 China

**Keywords:** Open gingival embrasures, Prevalence, Clear aligners, Interproximal enamel reduction, Attachments

## Abstract

**Background:**

The incidence of open gingival embrasures (OGE) in patients after fixed appliance treatment is relatively high, while there are no detailed reports on patients after clear aligner therapy. Also, no clinical studies with large sample size have investigated whether interproximal enamel reduction (IPR) can actually avoid OGE. The purpose of this study was to determine the prevalence of OGE in adults after clear aligner therapy and to investigate the risk of OGE associated with IPR treatment and attachment design, focusing on the amount and distribution in mandibular anterior teeth.

**Methods:**

Pre-treatment and post-treatment intraoral frontal photographs of 225 non-extraction patients were evaluated retrospectively for the occurrence and severity of OGE. The amount of IPR and the number of attachments in the anterior teeth from subjects after screening were recorded according to the first version of clear aligner software (Clincheck, San Jose, USA) and clinical medical documents. Logistic regression analysis was performed to identify the factors contributing to OGE.

**Results:**

The incidence of OGE in non-extraction patients after clear therapy between maxillary and mandibular central incisors was 25.7% and 40.3%, respectively. IPR was not associated with the occurrence of OGE but was associated with severity (*P* < 0.05). The number of attachments in the anterior teeth or central incisors was significantly related to the incidence of OGE (*P* < 0.05) but was not associated with severity.

**Conclusion:**

A high rate of OGE occurs after clear aligner therapy. Clinicians should be aware of the application of IPR and the design of attachments during clear aligner therapy.

## Background

Aesthetics after orthodontic treatment is an indispensable and important goal, in addition to dentition stability and good functional occlusion. A common aesthetic problem induced by orthodontic treatment is OGE, which could also lead to the retention of food debris and then adversely affect the health of the periodontium [1]. The incidence of OGE between the central incisors is known to be relatively high, more than one-third of adult patients have OGE after fixed appliance treatment [1, 2].

Clear aligners are increasingly popular due to their excellent aesthetics, their removability, and their easy-to-clean features; these properties may be better for periodontal health than fixed appliances [3]. However, some researchers have reported different viewpoints in that clear aligners tend to accumulate plaque at the marginal gingiva [4, 5] and have excessive initial stress [6], which are more likely to damage periodontal health and cause OGE. Evidence supports the use of clear aligners as an alternative to fixed appliances in patients with mild-to-moderate malocclusion, but not in severe cases [7]. Consequently, non-extraction patients make up the majority of invisible orthodontic patients.

Previous studies of OGE all focused on fixed appliances [1, 2, 8], and most of them discuss the incidence of OGE without distinguishing the type of extraction. OGE between the central incisors after orthodontic treatment has been reported in 22%—38% of patients with different types of extraction [1, 2], which increased to 68% in patients experiencing the extraction of a mandibular incisor [8]. Different types of tooth extraction correspond to different degrees of soft tissue remodeling; thus, the incidence of OGE can be completely different. The current research relating to the incidence of OGE in different types of tooth extraction is incomplete and remain unclear. Unfortunately, there are no detailed reports on the incidence of OGE in patients with clear aligners, let alone discussing a specific type of tooth extraction.

Research on the risk factors related to OGE has involved periodontal response, the movement of teeth, bone height, the distance from the alveolar bone crest to the interproximal contact point, tooth shape, and root angulations [1, 2, 8], which can be divided into anatomical characteristics of the teeth and alveolar bone, the characteristics of tooth movement and the patient's individual conditions. Clear aligners have different orthodontic accessories and biomechanics when compared to fixed appliances. Previous research has failed to investigate the specific predisposing factors of OGE in invisible orthodontics. The attachments of clear aligners serve as necessary auxiliary device which transfers the force from the aligners to the teeth. Aligner attachments control the direction, amount, and application point of orthodontic force, which arises from the intentional predetermined mismatching between the aligner and the attachments on these teeth [9]. Furthermore, attachments increase the retention of clear aligners so that they become stressed when the aligner is removed and inserted [10–12]. The more frequently the aligner is removed and inserted, the more often the attachment is stressed [10]. The effects of the distribution and number of attachments on periodontium have yet to be elucidated. Recently, IPR has surpassed extractions as the most popular orthodontic procedure in cases of invisible non-extraction [13]. Correcting anterior crowding with IPR can avoid OGE because the ideal gingival apposition area can reduce or prevent retrusion of papillae [14, 15], yet no evidence from clinical trials with large sample size have addressed this specific outcome.

The aim of the present study was to investigate the incidence and severity of OGE in non-extraction patients in invisible orthodontics, and then examine the risk of OGE associated with IPR treatment and attachment design.

## Materials and methods

Over 2500 records of adult healthy patients who completed orthodontic treatment with clear aligners (Invisalign, Align Technology, USA) were reviewed from December 2019 to December 2022 in the Department of Orthodontics, Stomatological Hospital of the Air Force Military Medical University. Patients were selected based on the following criteria: (1) they were over 18 years-of-age at the start of orthodontic treatment; (2) patients without a history of periodontal disease or OGE at the beginning of treatment; (3) intraoral photographs were available for before and after treatment. The exclusion criteria are as follows: (1) disagreement of the severity of OGE by all examiners; (2) restorations between the central incisors before treatment; (3) history of previous orthodontic treatment.

**Fig. 1:**
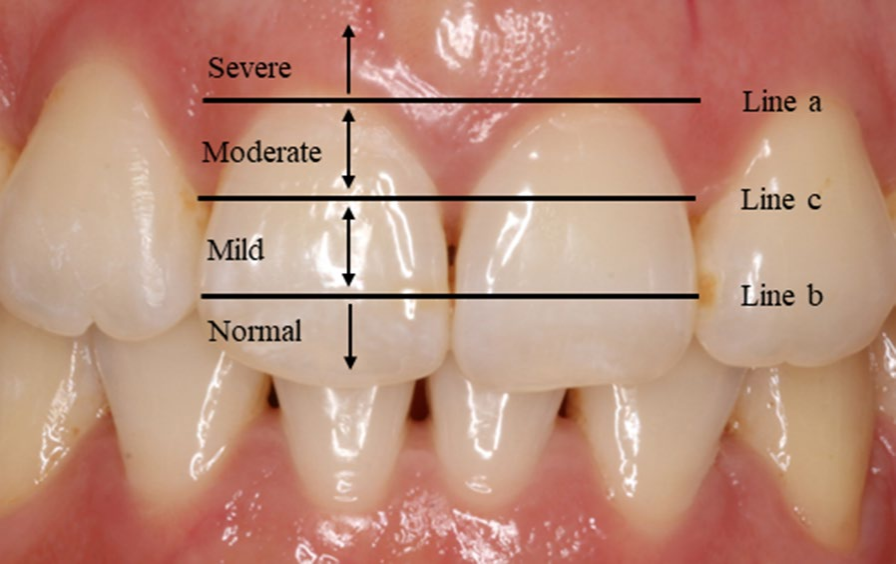
Classification of open gingival embrasures according to severity Three reference lines: **a** a tangent line to the highest gingival curvature of the crown, **b** a line passing through the most cervical contact point and **c** a line bisecting the distance between line **a** and line **b**.

To investigate the prevalence of OGE, frontal intraoral photographs taken before each therapy session and after the final treatment were evaluated by two orthodontists and one periodontist. The presence and severity of OGE between the maxillary central incisors and mandibular central incisors were evaluated with the Jemt index (Fig. 1), and the severity of OGE (normal, mild, moderate and severe) was determined [2, 16] as follows. Eighteen subjects were excluded because of disagreement. Ultimately, the final sample comprised 226 non-extraction patients.

- *Normal* Interdental papilla fills the embrasure space to the apical extent of the interdental contact point/area.
- *Mild* The tip of the interdental papilla lies between line b and line c.
- *Moderate* The tip of the interdental papilla lies between line a and line c.
- *Severe* The tip of the interdental papilla lies apical to line a.

Power calculations assumed a 40% prevalence of attachments in anterior teeth. At a ratio of 1:1, 40 controls and 40 cases were at least required for detection of an odds ratio of 5 with 90% power at a 5% significance level. Then, we selected a subsample of 125 subjects without tooth extraction (106 women and 19 men; mean age, 25.2 ± 6.7 years; mean treatment duration, 27.5 ± 12.2 months) with clinical medical records and clear aligner software (Clincheck, San Jose, USA). The malocclusion of these subjects: Class I (65 patients), Class II (48 patients) and Class III (12 patients). Selection was based on the following exclusion criteria: 0 mm ≤ anterior crowding ≤ 3 mm [17], lingual attachments in the anterior teeth and a treatment duration (of the first version) ≤ 24 months. The collected data were analyzed to assess the association of IPR and attachments in anterior teeth with OGE.

The amount and position of IPR, and the number and locations of attachment in the mandibular anterior teeth were recorded for each patient from the first version of the invisible treatment protocol by utilizing Clincheck software and analyzing clinical medical records. At the same time, intraoral photographs after the first version of treatment were collected to observe the occurrence and severity of OGE. The amount of IPR detected by Clincheck software corresponded with the amount of enamel removed in vivo [18]. The amount of IPR and the number of attachments were classified into categorical variables based on clinical practical application and available clinical reference [19]. The protocol was approved by the Ethical Committee of the Stomatological Hospital of the Air Force Military Medical University. Written informed consent was obtained from each participant.

### Statistical analysis

The agreement and reproducibility among raters were determined by using Fleiss kappa statistic. Categorical variables were described as frequency rates and percentages. Continuous variables were described as mean and standard deviation (SD) when variables were normally distributed, or median and interquartile range (IQR) when variables were not normally distributed. The Chi-squared test or Fisher’s exact test was used to compare categorical variables and the independent t test was used to compare continuous variables. The possible risk factors for OGE were analyzed by univariate logistic regression analysis. Variables that were considered significant (*P* < 0.1) were then analyzed by multivariate logistic regression analysis to identify independent risk factors for OGE. Statistical analyses were performed using IBM SPSS version 22.0 (IBM Corp, Armonk, New York, USA). All tests were two-sided and *P* < 0.05 was considered statistically significant.

## Results

The overall kappa statistic was 0.953, indicating almost perfect agreement (*P* < 0.001). Of the 226 non-extraction patients after invisible orthodontics, the incidence of OGE between maxillary the central incisors and the mandibular central incisors was 25.7% and 40.3%, respectively. The moderate OGE in the mandible accounted for 10.2% of the total number of patients; this was higher than the 5.8% in the maxilla. Severe OGE was not observed in either the maxilla or mandible. Similar trends were observed in 125 subjects after the first version of treatment (26.4% and 40.8% OGE in the maxilla and mandible, 6.4% and 11.2% moderate OGE in the maxilla and mandible, respectively, Table 1). Subsequently, we investigated the association of IPR and attachments in the mandibular anterior teeth with OGE.

**Table 1:** Incidence and severity of open gingival embrasures in non-extraction patients

	Non-occurrence	Occurrence		
		Mild	Moderate	Severe
After clear therapy				
Maxilla (n = 226)	168 (74.3)	45 (19.9)	13 (5.8)	0
Mandible (n = 226)	135 (59.7)	68 (30.1)	23 (10.2)	0
After the first version of treatment				
Maxilla (n = 125)	92 (73.6)	25 (20)	8 (6.4)	0
Mandible (n = 125)	74 (59.2)	37 (29.6)	24 (19.2)	0

A comparison between the non-occurrence and occurrence groups in 125 non-extraction patients after the first version of treatment is given in Table 2. Age, treatment duration, gender and malocclusion did not differ between the two groups. Surprisingly, the amount and distribution of IPR were similar in patients with OGE and patients without OGE. It is worth noting that the occurrence groups had more patients with attachments in the anterior teeth when compared to the non-occurrence groups (98.0% *vs* 82.4%, *P* < 0.05); and 29 (56.9%) patients in the OGE groups had over two attachments in the anterior teeth. Similarly, patients in which both of the central incisors had attachments accounted for more patients in the occurrence groups than the non-occurrence groups (13.7% *vs* 1.4%, *P* < 0.05). A severity-based assessment of OGE is shown in Table 3. Moderate OGEs mainly featured patients without IPR between the central incisors while one half of patients with mild OGE were those without IPR between the central incisors (85.7% *vs* 51.4%, *P* < 0.05).

**Table 2:** Comparison of open gingival embrasures between the non-occurrence and occurrence groups in the mandible

Variables	Non-occurrence (*N* = 74)	Occurrence (*N* = 51)	*P* value
Age, years	24.5 ± 7.3	26.4 ± 5.6	0.113
Treatment duration, months	27.1 ± 12.5	28.1 ± 12.0	0.692
*Gender*			0.374
Female	61 (82.4)	45 (88.2)	
Male	13 (17.6)	6 (11.8)	
*Malocclusion*			0.864
Class I	37 (50.0)	28 (54.9)	
Class II	30 (40.5)	18 (35.3)	
Class III	7 (9.5)	5 (9.8)	
*IPR in anterior teeth*			0.199
0 mm	29 (39.2)	18 (35.3)	
0–2 mm	17 (23.0)	19 (37.3)	
≥ 2 mm	28 (37.8)	14 (27.5)	
*IPR in incisors*			0.683
0 mm	34 (45.9)	21 (41.2)	
0–1 mm	18 (24.3)	16 (31.4)	
≥ 1 mm	22 (29.7)	14 (27.5)	
*IPR in central incisors*			0.653
0 mm	42 (56.8)	31 (60.8)	
> 0 mm	32 (43.2)	20 (39.2)	
*Attachments in anterior teeth*			0.021*
0^a^	13 (17.6)	1 (2.0)	
1-2^b^	29 (39.2)	21 (41.2)	
> 2^b^	32 (43.2)	29 (56.9)	
*Attachments in incisors*			0.222
0	45 (60.8)	31 (60.8)	
1–2	25 (33.8)	13 (25.5)	
> 2	4 (5.4)	7 (13.7)	
*Attachments in central incisors*			0.022*
0^a^	66 (89.2)	40 (78.4)	
1^a^	7 (9.5)	4 (7.8)	
2^b^	1 (1.4)	7 (13.7)	

Data are given as mean ± SD or *N* (%)

*IPR* interproximal enamel reduction

*P* value < 0.05 was considered significant (*) given in Bold

**Table 3:** Comparison of open gingival embrasures between the mild and moderate groups in the mandible

Variables	Mild (*N* = 37)	Moderate (*N* = 14)	*P* value
Age, years	26.1 ± 5.5	27.4 ± 6.0	0.477
Treatment duration, months	24.1 ± 9.3	28.9 ± 12.4	0.385
*Sex*			0.327
Female	34 (91.9)	11 (78.6)	
Male	3 (8.1)	3 (21.4)	
*Malocclusion*			0.812
Class I	19 (51.3)	9 (64.3)	
Class II	14 (37.8)	4 (28.6)	
Class III	4 (10.8)	1 (7.1)	
*IPR in anterior teeth*			0.527
0 mm	12 (32.3)	6 (42.9)	
0–2 mm	13 (35.1)	6 (42.9)	
≥ 2 mm	12 (32.4)	2 (14.3)	
*IPR in incisors*			0.091
0 mm	15 (40.5)	6 (42.9)	
0–1 mm	9 (24.3)	7 (50.0)	
≥ 1 mm	13 (35.1)	1 (7.1)	
*IPR in central incisors*			0.025*
0 mm ^a^	19 (51.4)	12 (85.7)	
> 0 mm ^b^	18 (48.6)	2 (14.3)	
*Attachments in anterior teeth*			0.820
0	1 (2.7)	0 (0)	
1–2	16 (43.2)	5 (35.7)	
> 2	20 (54.1)	9 (64.3)	
*Attachments in incisors*			0.607
0	21 (56.8)	10 (71.4)	
1–2	11 (29.7)	2 (14.3)	
> 2	5 (13.5)	2 (14.3)	
*Attachments in central incisors*			0.637
0	28 (75.7)	12 (85.7)	
1	4 (10.8)	0 (0)	
2	5 (13.5)	2 (14.3)	

Data are given as mean ± SD or *N* (%)

*IPR* interproximal enamel reduction

*P* value < 0.05 was considered significant (*) given in Bold

The difference of superscript letter (a & b) means significantly different between groups

Univariate and multivariate analysis are shown in Table 4. In the univariate analysis, IPR was not associated with the occurrence of OGE. Attachments that existed in the anterior teeth or central teeth were associated with OGE between the mandibular central incisors. In multivariate analysis, 1–2 attachments in the anterior teeth (odds ratio [OR]: 9.555), > 2 attachments in the anterior teeth (OR: 9.578), and 2 attachments in the central incisors (OR: 9.501) were all independent risk factors associated with OGE.

**Table 4:** The relationship between risk factors and OGE by univariate and multivariate analysis

Variables	Univariate analysis			Multivariate analysis		
	OR	95% CI	*P* value	OR	95% CI	*P* value
Age	1.045	0.221–1.772	0.377			
Treatment duration	1.007	0.989–1.104	0.116			
Sex	0.626	0.973–1.042	0.688			
*Malocclusion*						
Class I	Reference					
Class II	0.793	0.370–1.701	0.551			
Class III	0.944	0.271–3.289	0.928			
*IPR in anterior teeth*						
0 mm	Reference					
0–2 mm	1.801	0.747–4.340	0.190			
≥ 2 mm	0.806	0.337–1.923	0.626			
*IPR in incisors*						
0 mm	Reference					
0–1 mm	1.439	0.606–3.420	0.410			
≥ 1 mm	1.030	0.435–2.442	0.946			
*IPR in central incisors*						
0 mm	Reference					
> 0 mm	0.847	0.410–1.751	0.654			
*Attachments in anterior teeth*						
0	Reference					
1–2	9.414	1.141–77.652	0.037*	9.555	1.157–78.923	0.036*
> 2	11.781	1.450–95.738	0.021*	9.578	1.156–79.368	0.036*
*Attachments in incisors*						
0	Reference					
1–2	0.755	0.335–1.699	0.497			
> 2	2.540	0.685–9.432	0.163			
*Attachments in central incisors*						
0	Reference					
1	0.943	0.260–3.424	0.929	0.776	0.209–2.876	0.704
2	11.550	1.370–97.365	0.024*	9.501	1.081–83.522	0.042*

*IPR* interproximal enamel reduction

*P* value < 0.05 was considered significant (*) given in Bold

## Discussion

In addition to esthetics problems, OGE is also associated with various functional implications, such as gingival inflammation, poor gingival health and the loss of integrity [20]. In our study, OGE occurred in 25.7% of maxillary and 40.3% of mandibular central incisors after clear aligner therapy. IPR was not associated with the occurrence of OGE but was associated with severity. The number of attachments in the anterior teeth or central incisors was significantly related to the incidence of OGE but this was not associated with severity. Furthermore, the incidence and severity of OGE in the mandibular was higher and more severe than in the maxillary. The risk of aesthetic loss caused by clear aligners calls for clinicians’ attention.

The incidence of OGE between the incisors in non-extraction patients after clear therapy ranged from 25.7–40.3%, while other researchers previously reported that the incidence of OGE in both non-extraction and extraction patients was 22–36% after the fixed appliance treatment [2]. The incidence of OGE seems to be higher in extraction patients than in non-extraction patients because of the greater difficulty and more retraction of teeth [21–23]. Consequently, we conjectured that the incidence of OGE in patients with clear aligners is higher than that of patients with fixed appliances. However, more precise clinical studies are needed to confirm this conjecture.

Clear aligners appear to be associated with periodontal risk for several reasons. Firstly, the initial stress from clear aligners is approximately 50 to 500 times more than that of fixed orthodontic loading [5], and the instantaneous higher-stress may have a certain impact on the periodontal tissues. The excessive initial stress of the clear aligners was demonstrated in rats that received a clear plastic appliance; hyalinization of the PDL was evident in the areas of compression as early as day 1 [24]. In the meanwhile, the labial movement of the anterior teeth in non-extraction cases induced alveolar bone loss and jeopardized periodontal health, especially in adults [23, 25], which suggest a risk for non-extraction patients. Due to the advantages of clear aligners, such as molar distalization, non-extractive orthodontics may be pursued excessively in borderline extraction cases. Additionally, high active forces and levels of compression, which could cause net bone loss and extensive gingival recession, were identified in the marginal tissues during tipping movements and intrusion [23]. Unfortunately, torque control in clear aligners is not as effective as in braces [26]. Therefore, orthodontists need to reconsider the biomechanics of clear aligners and determine the applicable population of clear aligners cautiously.

Researchers have also found that OGE may appear when the distance from the contact point to the alveolar crest is > 5 mm [27]; thus, IPR is an effective means with which to relocate the contact point [14, 15], although there is no clinical evidence that IPR can prevent the occurrence of OGE. Interestingly, in the present study, we did not find a relationship between IPR and the occurrence of OGE, but we conjectured that IPR could reduce the severity of OGE. Further studies with larger sample size are required to confirm this conjecture through multinominal regression analysis. Perhaps preventive IPR is lack of accuracy, and the amount of preventive IPR is inadequate. The contact points at the beginning of treatment are usually different from the contact points after treatment because the root angle of the untreated anterior teeth is abnormal and teeth may overlap due to crowding [20]. Preventive IPR cannot directly target the position of contact points after treatment which are really related to OGE. Also, the amount of IPR of no more than half the enamel coating’s original thickness was recommended to avoid proximity to the dentin, thus indicating that the amount of IPR at an interproximal gap is finite and may not be sufficient to prevent the occurrence of OGE [28]. In contrast, as a means of treatment, IPR can be accurately located and the amount of IPR can be adjusted based on comprehensive consideration. IPR is generally proposed in treatment programs to provide space to solve crowding or repair abnormal tooth morphology [15]. In addition to being used as a therapeutic method, IPR is associated with the release of anterior teeth adjacent points, and incomplete release of anterior teeth will hinder tooth movement, thus leading to adverse stress transfer among teeth. The categorical and measurement data of IPR in this paper reflects not only the amount at a specific interdental gap, but also the extensiveness of its distribution. Unfortunately, we did not identify a relationship between IPR in anterior teeth and OGE in the central incisors. The relationship between the release of the interproximal space by IPR and force conduction among teeth requires further in-depth studies. Above all, for inexperienced clinicians, additional preventive IPR may be unnecessary but regular interdental release is necessary, clinicians only need to carry out IPR according to specific clinical needs. When OGE occurs, a small amount of IPR (but applied frequently) as a treatment method could be carried out, and combined with other methods like the intrusion to improve OGE.

There are two types of force transmitted from the attachments to the periodontium: active force [9] and removal stress [29]. The odds ratio of subgroups in which the patients had attachments on both central incisors was 9.501, indicating that the occurrence of OGE was 9.501-fold higher when compared to the subgroup of patients who did not have attachments in the central incisors. For patients in which both central incisors had attachments, the two types of forces from attachments are greater than those in patients without attachments in the incisors. Furthermore, the number of attachments reflects the complexity of tooth movement [30]. In order to exclude other interfering factors as accurately as possible, the thickness and material of the diaphragm remained the same [8], and non-extraction patients were selected for this study so as to unify and simplify tooth movement types as far as possible. In addition, our results showed that attachments on the canines had a greater effect on OGE between the central incisor than attachments on the lateral incisor. Because our study did not distinguish between optimal attachments and the traditional attachments more commonly used on canine teeth, the removal forces may be greater for the retainer attachments in canine teeth. Besides, in two-step intrusion, attachments in the canines can also act as anchorage to the intrusion of incisors; this may be related more closely to the movement of central incisors. Nevertheless, further biomechanical investigation of the relationship between the distribution of attachments and OGE is needed. The features of attachments not only help control tooth movement but also promote optimal biological responses. Consequently, in addition to tooth movement needs, the design of the attachment should also consider the periodontal conditions of the anterior teeth.

We found that the incidence of OGE at the end of the first version of treatment was similar to that after complete treatment, thus suggesting that the data on IPR and attachments collected from subgroups was representative. Age, the duration of treatment and gender were not significantly related to the occurrence and severity of OGE. As patients with periodontitis were excluded from this study, the effect of age may have been underestimated. Since we only selected non-extraction patients, the treatment difficulty was similar, and the treatment time was not statistically different. Moreover, due to the aesthetic outcomes associated with clear aligners, most patients are female; there was no significant difference between the groupings with respect to gender.

To the best of our knowledge, this study is the first to assess the prevalence of OGE after clear aligner therapy. We attempted to objectively investigate the relationship between OGE and contributing factors that are unique to clear aligners, such as attachments. Furthermore, this article was an initial clinical study to explore that whether IPR can prevent OGE. With the increasing popularity of clear aligners, more studies focusing on clear aligners are necessary in the future.

### Limitations

There are some limitations in this study that need to be considered. Additional variables, such as crowding and alveolar bone height, could not be included in the regression model due to numerical insufficiency; because the number of variables entered into a logistic regression model increases, the stability of the model decreases. For this reason, we utilized stricter inclusion and exclusion criteria to minimize the risk of bias as much as possible. Because there may have been more than one version of Clincheck software for each patient, we could only evaluate the number of attachments according to the first version, select the first version of treatment that was longer than 24 months, and match corresponding intraoral photographs. Due to the limitations of the retrospective study, we could only assess the prevalence of OGE from frontal intraoral photographs, the periodontal index could not be obtained. In addition, the condition of dentition gathered from the overlap of the intraoral scan model before and after treatment would be more accurate and instructive for the research in invisible orthodontics. Our study only evaluated the central incisors because of distortion and poor reproducibility of the lateral or canine areas in intraoral photographs. Additional studies that evaluate the incidence of different patients following tooth extraction after clear therapy and the biotype of the gingiva would be more meaningful and more instructive for clinical practice.

## Conclusions

The incidence of OGE in non-extraction patients in adults after clear aligner therapy between the maxillary and mandibular central incisors was 25.7% and 40.3%, respectively. IPR cannot prevent the occurrence of OGE. The number of attachments in the anterior teeth or central incisors is positively correlated with the incidence of OGE between the central incisors.

## Data Availability

The raw data are present in our university clinic.

## Abbreviations

OGE: Open gingival embrasures
IPR: Interproximal enamel reduction
